# Music in the eye of the beholder: a pupillometric study on preferred background music, attentional state, and arousal

**DOI:** 10.1007/s00426-024-01963-8

**Published:** 2024-04-23

**Authors:** Luca Kiss, Bence Szikora, Karina J Linnell

**Affiliations:** 1 Department of Psychology, Goldsmiths University of London, 8 Lewisham Way New Cross, London, SE14 6NW UK; 2 London, United Kingdom

## Abstract

Although background music listening during attention-demanding tasks is common, there is little research on how it affects fluctuations in attentional state and how these fluctuations are linked to physiological arousal. The present study built on Kiss and Linnell (2021) - showing a decrease in mind-wandering and increase in task-focus states with background music - to explore the link between attentional state and arousal with and without background music. 39 students between the ages of 19–32 completed a variation of the Psychomotor Vigilance Task in silence and with their self-selected background music (music they would normally listen to during attention-demanding tasks). Objective arousal measures (pretrial pupil diameter and task-evoked pupillary responses) and subjective attentional state measures (mind-wandering, task-focus, and external-distraction states) were collected throughout the task. Results showed a link between attentional state and arousal and indicated that background music increased arousal. Importantly, arousal mediated the effect of music to decrease mind-wandering and increase task-focus attentional states, suggesting that the arousal increase induced by music was behind the changes in attentional states. These findings show, for the first time in the context of background music listening, that there is a link between arousal and attentional state.

## Introduction

Even though background music listening during simple tasks is widespread (e.g., Kiss & Linnell, 2023), it is still not clear how it affects attention and performance (see Dalton & Behm, 2007; Kämpfe et al., 2011; Küssner, 2017, Mendes et al., 2021; Schwartz et al., 2017, for reviews). Findings are contradictory with some suggesting a positive, some a negative, and some no effect of the music (see e.g., Kämpfe et al., 2011). Seemingly contradictory findings in the literature could be a consequence of a variety of factors, including (1) differences in the type of task performed while listening to music in the background, (2) differences in parameters of the music, and (3) differences between individuals (for further discussion of these factors, see Gonzalez & Aiello, 2019). Differences in these factors (namely the task, the music, and the individual) can result in background music affecting attention and performance in opposite directions (e.g., Gonzalez & Aiello, 2019). The impact of all of these factors could be mediated by differences in physiological arousal, given that the ‘arousingness’ of both tasks (e.g., Baron, 1986) and music (e.g., Lynar et al., 2017; see also Husain et al., 2002; Thompson et al., 2001; Schellenberg, 2005; Schellenberg & Hallam, 2005; Schellenberg et al., 2007) can vary, as can individual levels of baseline arousal (Cassidy & McDonald 2007; Küssner, 2017).

The arousal theory highlights that there is an inverted-U shaped relationship between arousal and performance such that only an intermediate arousal level is linked to optimal performance and both lower than optimal (i.e., sub-optimal) and higher than optimal (i.e., supra-optimal) arousal levels result in decreased performance (Yerkes & Dodson, 1908). Recent studies have linked sub- and supra-optimal arousal levels to attentional lapses that can take two forms, namely mind-wandering or external-distraction states (e.g., Unsworth & Robison, 2016). As highlighted in a recent theoretical model, both of these states can either be linked to arousal levels that are lower or higher than optimal, and which attentional lapse one experiences has been proposed to depend on whether one’s attention is oriented to the external or internal environment: when attention is oriented externally, this results in external-distraction states, encompassing on the one hand *prepotent responses* when arousal is sub-optimal or on the other hand *stimulus sensitivity* when arousal is supra-optimal; when attention is oriented to internal thoughts and feelings this results in mind-wandering states, and on the one hand *mind-wandering* when arousal is sub-optimal or on the other hand *racing thoughts/rumination* when arousal is supra-optimal (Lenartowicz et al., 2013; see also Unsworth & Robison, 2018, for a study on low versus high arousal mind-wandering states). Attentional lapses have been found to be underpinned by the locus coeruleus–norepinephrine system (LC–NE; Cohen et al., 2004). The LC is a neuromodulatory nucleus in the brain stem that projects norepinephrine to the neocortex and mediates effects of arousal on attention and performance (Berridge & Waterhouse, 2003). Specifically, fluctuations in baseline LC activity correspond to fluctuations in attentional state such that when baseline LC activity, or arousal, is either lower or higher than optimal, performance is poorer (Aston-Jones & Cohen, 2005) and attentional lapses are experienced (e.g., Unsworth & Robison, 2016, 2018).

Given that background music can increase arousal (e.g., Burkhard et al., 2018; Caldwell & Riby, 2007; Cassidy & MacDonald, 2007; Nantais & Schellenberg, 1999; Nguyen & Grahn, 2017; North & Hargreaves, 1999) and simple tasks tend to be under-arousing in themselves (e.g., Baron, 1986; Fischer et al., 2008; Gonzalez & Aiello, 2019), music listened to in the background during simple tasks can be expected to increase arousal to an intermediate level optimal for task-focused attention and performance (Kiss & Linnell, 2021). This idea has previously been explored with subjective measures of attentional state by Kiss and Linnell (2021) who found that task-focus states increased, and mind-wandering states decreased, while participants performed a sustained attention task with preferred background music compared to silence. Based on work by Unsworth and Robison (2016) showing that during a sustained attention task task-focus states were associated with optimal arousal levels and mind-wandering states with sub-optimal arousal levels, these findings with subjective attentional state (Kiss & Linnell, 2021) are compatible with music increasing arousal to a more optimal level for task-focus. Nevertheless, it is important to collect objective measures of arousal to validate the interpretation of past findings on attentional state and background music. Thus, the overall aim of the current study was to extend findings by Kiss and Linnell (2021) and explore the effect of preferred background music on, simultaneously, subjective reports of attentional state and an objective measure of arousal.

Good potential candidates for objectively measuring changes in arousal and attentional state are the linked psychophysiological markers of pretrial pupil diameter, the size of the pupil before each trial of a task, and task-evoked pupillary responses (TEPRs), the dilation of the pupil relative to baseline levels in response to the cognitive demands of each trial of a task (e.g., Beatty, 1982; Franklin et al., 2013; Grandchamp et al., 2014; Mittner et al., 2014; Kang et al., 2014; Smallwood et al., 2011; Unsworth & Robison, 2016, 2017, 2018; Unsworth et al., 2018). Indirect, correlational relationships between pupil diameter/TEPR and the functioning of the LC-NE arousal system have long been established, suggesting that pupillometric measures can index modes of the LC. Specifically, intermediate LC activity - where attention is focused and performance is best - is linked to intermediate pretrial pupil diameter and higher TEPR; on the other hand, lower and higher LC activity - linked to lower and higher pretrial pupil diameters respectively - are both linked to lower TEPR (see, e.g., Aston-Jones & Cohen, 2005; Gilzenrat et al., 2010; Rajkowski et al., 1993). Although pupillometric evidence for the inverted-U model of arousal and performance has been surprisingly elusive (Van den Brink et al., 2016), recent studies linking pupillometric measures directly to subjectively reported attentional state have provided evidence for the model. For example, Unsworth and Robison (2016) found that pupil diameter and TEPR could accurately track fluctuations in attentional state and distinguish mind-wandering, task-focus, and external-distraction states, and were consistent with prior research on the LC-NE and on the modes of functioning of the LC. Specifically, task-focus states were reliably associated with intermediate pupil diameter and higher TEPR, mind-wandering states reliably associated with lower pupil diameter and lower TEPR, and external-distraction states with higher pupil diameter and lower TEPR.

Although research from the wider music literature focusing on pupillometry and music listening without performance of a concurrent task provides evidence for an effect of music on pupillary responses (e.g., Bianco, Gold, Johnson, & Penhune, 2019; Bishop, Refsum, & Laeng, 2021; Bowling, Acochea, Hove, & Fitch, 2019; Gingras et al., 2015; Jagiello, Pomper, Yoneya, Zhao, & Chait, 2019; Laeng et al., 2016; Liao, Makoto, Kashimo, & Furukawa, 2018; Tervaniemi, Makkonen, & Peixin, 2021), research on pupillometry and *background music* listening (i.e., with concurrent performance of a task) is limited (see, e.g., Johansson, Holmqvist, Mossberg, & Lindgren, 2012, for a study on pupillometry and reading comprehension with and without background music). To our knowledge, there is only one study (a Master’s thesis) to-date on pupillometry and background music listening during a simple attention-demanding task (Tamaliunaite, 2017). In their study, Tamaliunaite (2017) explored the link between background music listening - varying in tempo and percussiveness - and pupillometric as well as subjective measures of arousal during performance of the colour-word Stroop test, used to measure selective attention. Their results showed that background music increased pretrial pupil diameter suggesting an increase in arousal, but background music did not improve performance on the Stroop task. Tamaliunaite (2017) did not however measure attentional state and did not use participants’ preferred background music.

Thus, to expand past findings, in addition to collecting pupillometric data, in the current study subjective reports of attentional state were collected during performance of a simple sustained attention/continuous-performance task. To our knowledge, this is the first study to-date to collect both pupillometric data and direct reports of attentional state with and without background music. Collecting both pupillometric data and direct reports of attentional state allowed us to examine, for the first time, whether arousal indeed mediates the effect of background music on attentional state. Here we used participants’ preferred or self-selected background music to increase ecological validity and account for individual differences in music preference and baseline arousal (Cassidy & MacDonald, 2009; Darrow et al., 2006; Ünal et al., 2012, 2013). Because baseline arousal level varies across individuals and depends on factors such as personality (e.g., Cassidy & MacDonald, 2007; Furnham & Allass, 1999; Salame & Baddeley, 1982), and because music preference also varies across people, the ‘arousingness’ and the qualities of the music required to achieve optimal arousal and performance can also be expected to vary across individuals.

The aim of the present study was to explore the effect of preferred background music on objectively measured arousal as indexed by pupil responses, including pretrial pupil diameter and TEPR. Moreover, we explored the link between objectively measured arousal and subjectively reported states of mind-wandering, task-focus, and external-distraction with and without background music, and whether arousal mediates the link between background music and attentional states. This study formed part of a larger study the first part of which - focusing only on subjective attentional states - was reported in Kiss and Linnell (2021). Using the same dataset to further explore the effects of background music on attentional state using objective as well as subjective measures meant that we could directly compare subjective and objective results and test the applicability of the inverted-U arousal model, and work linking it to attentional state, to background music listening. Focusing only on subjectively reported attentional states (here termed thought-probe responses) in Kiss and Linnell (2021) did not make it possible to test whether fluctuations in attentional state reported in the context of background music listening are compatible with the inverted-U arousal model, as arousal was not directly measured. With the addition of pupillary responses in the present study, it was possible to directly measure arousal levels with and without background music. This, in turn, enabled us to explore whether the decrease in mind-wandering and increase in task-focus states reported in Kiss and Linnell (2021) is indeed mediated by an increase in arousal, which was the final aim of the current study.

With the first hypothesis, we focused on the link between arousal and fluctuations in attentional state: this hypothesis was based on the inverted-U arousal model linking arousal to performance and on work by Unsworth and Robison (2016) showing that, during the Psychomotor Vigilance Task measuring sustained attention, an intermediate arousal level was linked to task-focus states while lower and higher arousal levels were linked to mind-wandering states and external-distraction states respectively. We expected that, during a similar sustained attention task used here (a variation of the Psychomotor Vigilance Task), task-focus states should be linked to an intermediate arousal level and mind-wandering states and external-distraction states to lower and higher arousal levels respectively. We also focused on time on task effects on arousal, similarly to Unsworth and Robison (2016), and expected arousal to decrease with an increase in time spent on the task.

Given that preferred background music increased task-focus and decreased mind-wandering states in Kiss and Linnell (2021), and if task-focus states are indeed associated with optimal arousal and mind-wandering states with sub-optimal arousal level, with the second hypothesis we expected that music would be accompanied by an increase in physiological arousal, as indexed both by pretrial pupil diameter and TEPR.

Finally, we conducted an exploratory mediation analysis, expecting arousal to mediate the effect of background music on task-focus and mind-wandering states. To test this idea, with the final, third hypothesis we hypothesised that arousal should mediate the impact of background music on the balance of mind-wandering and task-focus states. This third hypothesis on mediation effects is also relevant to the mood-and-arousal hypothesis which states that music listening leads to an increase in positive mood and arousal which in-turn increases task-performance (Husain et al., 2002; Schellenberg, 2005; Schellenberg & Hallam, 2005; Schellenberg et al., 2007; Thompson et al., 2001).

## Method

### Design

This study was part of a larger study the first part of which - focusing only on subjective attentional states - was reported in Kiss and Linnell (2021). As already reported in Kiss and Linnell (2021), this study had a within-subjects design; all participants completed the task in silence and with background music. The independent variables were music-present/absent (music P/A), time-on-task (block 1, 2, 3, 4, and 5) and attentional-state category (mind-wandering, task-focus, external distraction). The order of the music conditions was counterbalanced, meaning that every second participant completed the task in the same order. This resulted in 19 participants completing the task in music-present followed by music-absent order and 20 participants in music-absent followed by music-present order. Here the main dependent variables were pretrial pupil diameter and task-evoked pupillary responses (TEPRs).

### Participants

Participants were those who completed the study reported in Kiss and Linnell (2021). They were students living in and around London who took part in the study on a voluntary basis in exchange for £5. Only students who normally listen to background music when performing attention-demanding tasks were included in the experiment. This inclusion criterion was supported by findings that people perform better in their preferred listening condition (Crawford & Strapp, 1994; Nantais & Schellenberg, 1999). Compared to the 40 participants in Kiss and Linnell (2021), here there were 39 participants in total as pupil data from the one of the participants was missing. Participants included 22 females and 17 males between the ages of 19 and 32 (*M* = 24, *SD* = 3.33).

### Materials

#### Preferred background music

As reported in Kiss and Linnell (2021), participants were asked to send a 30-min long playlist containing their preferred background music tracks to the experimenter prior to participation in the study. There were no restrictions on the music, but participants were asked to send a playlist they would normally listen to when performing an attention-demanding task. The individual tracks could be of any length (mean track duration was 215.57 s) but together the playlist had to cover the full 30 min of the music present session of the experiment. A few playlists were shorter than 30 min because some participants decided to listen to their chosen tracks in repeat, as they would normally do in real-life settings.

**Fig. 1:**
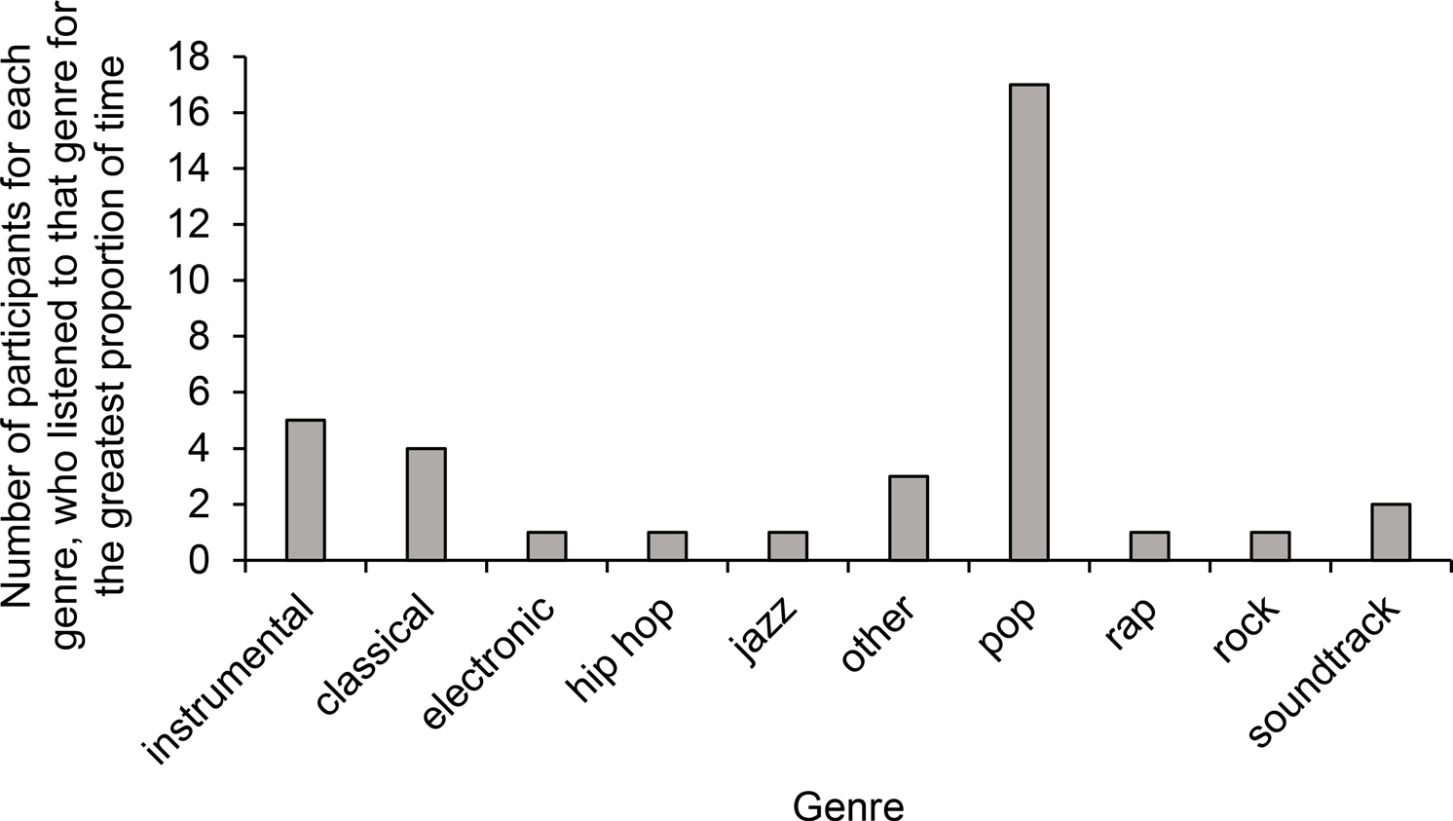
Each participant was assigned to the genre that made up the greatest proportion of time in their playlist. The graph represents the number of participants for each genre who listened to that genre for the greatest proportion of time. The ‘other’ category included tracks that could not be otherwise categorised, such as Japanese anime music, music for meditation, and some indie and folk tracks that did not clearly fit into a different main category. The ‘instrumental’ category included tracks that were primarily produced using musical instruments, such as remixes of original tracks, piano ‘lounge’ music, and ‘ambient’ music

Musicological data for each track were collected from the database of the digital music streaming service called Spotify, using the Spotify Web API endpoints (Spotify, 2018). This included data on tempo (overall tempo of a track in beats/minute; *M* = 112.56 BPM, *SD* = 32.37 BPM, range for individual tracks = 52.16–215.04 BPM), lyrics (i.e., a measure between 0.00 and 1.00 indicating the likelihood of a track containing any vocals at any given point during the track; tracks with a lower lyrics level were less likely to contain any vocal content; *M* = 0.59, *SD* = 0.57, range for individual tracks = 0.01–1.00), and genre. Genre categorisation for each track was based on Spotify’s main genre categories (Spotify, 2018); because there was no specific genre categorisation available for individual tracks, genres describing the artist and the album in which the track appeared were used. Specifically, the most frequently occurring main genre was chosen for each track. Tracks that only had subgenre labels and not a clear main genre assigned to them were placed in the ‘other’ category (see Fig. 1).

#### Sustained attention task

**Fig. 2:**
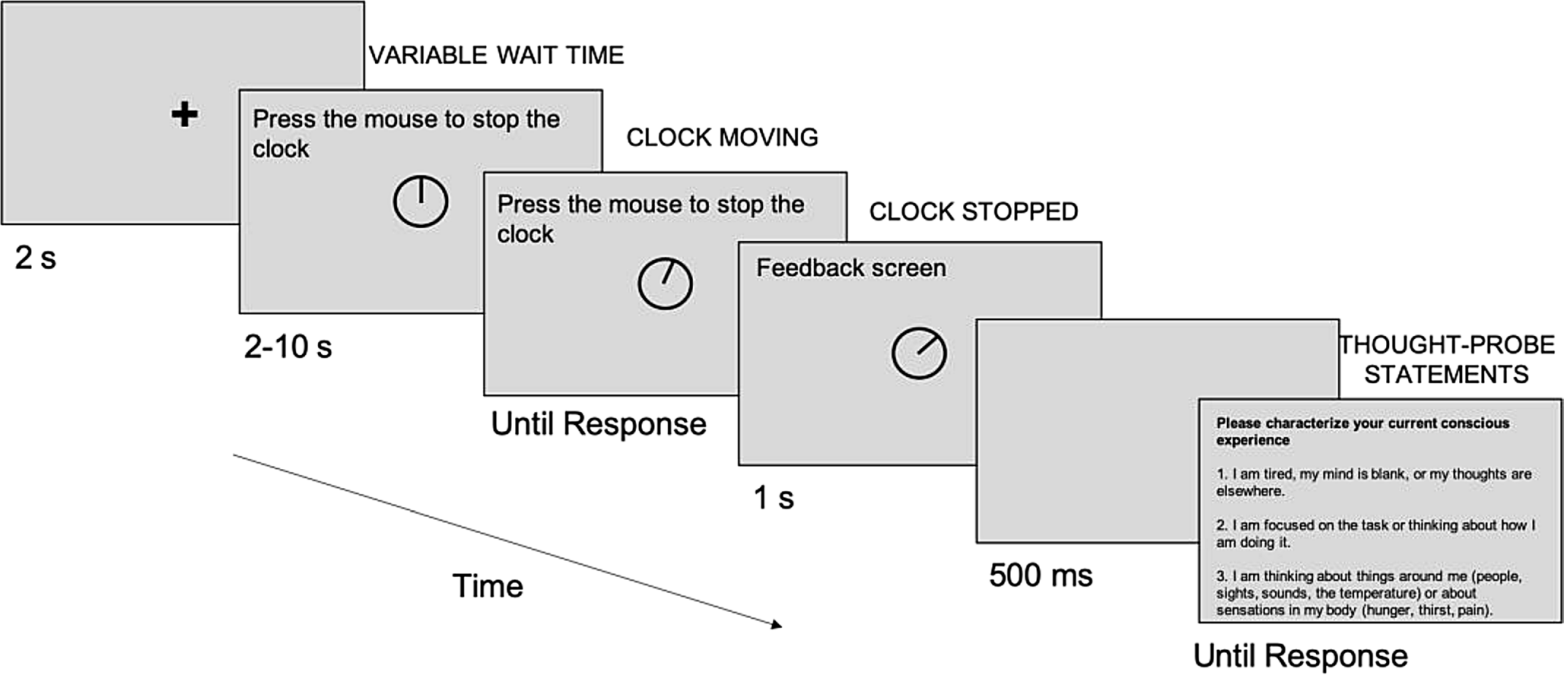
Schematic representation of a single experimental trial with a thought-probe after the trial

As reported in Kiss and Linnell (2021), the sustained-attention task was a variation of the Psychomotor Vigilance Task developed by Dinges and Powell (1985) which has long been used to measure sustained attention (Unsworth & Robison, 2016). As shown in Fig. 2, participants were first presented with a fixation cross in the middle of the screen on a grey background for 2 s. Then, they saw a clock without any numbers, specifically a black circle containing a clock-hand at the 12-o’clock position and, after a variable wait time (equally distributed from 2 to 10 s in 500 ms increments), the clock-hand started moving clockwise in a smooth analogue fashion. The task of the participants was to press the left mouse button as quickly as possible once the hand of the clock started moving. After the participant had pressed the mouse button, the clock remained on the screen for 1 s to provide feedback. Then, a 500-ms blank screen was presented, followed by either the next trial or a thought-probe. Participants completed 5 blocks, with 34 trials in each block, for both the music and no-music conditions. One block lasted approximately 6 min. Before starting the experiment, participants completed five practice trials to become familiar with the task.

During each block, participants were periodically presented with thought-probes. They were primed to respond to these probes by selecting the description of attentional state that best reflected their immediately preceding thoughts. They were presented with six thought-probes in every block of 34 trials, with the thought-probes randomly distributed across the block. Thought-probe descriptions, as specified below, were based on those used by Unsworth and Robison (2016).

Some alterations were made compared to previous research based on feedback from the participants in a pilot study reported in Kiss and Linnell (2021), to avoid ambiguous expressions that could refer to different concepts depending on one’s cultural background and understanding. More specifically, compared to the original five statements used in Unsworth and Robison (2016), only three statements were included in this study, namely those referring to mind-wandering, on-task thoughts/task-focus and external distraction, respectively (see the statements below). The term mind-wandering was left out of the phrasing of statement 1, and statements 2 and 3 - referring to task-focus and external distraction - were simplified.

“Please characterise your current conscious experience.

1. I am tired, my mind is blank, or my thoughts are elsewhere.
2. I am focused on the task or how I am doing it.
3. I am thinking about the things around me (people, sights, sounds, the temperature) or about sensations in my body (hunger, thirst, pain).”

### Apparatus and procedure

The current study was approved by the Psychology Department Ethics Committee at Goldsmiths, University of London on the 6th of June 2018. Participants were tested individually in the lab in a dark and quiet room (lights were switched off before the eye tracker calibration) and stimuli were presented on a 24-in. monitor with a 1920 × 1080 screen resolution equipped with an Eyelink 1000 eye tracker. To collect pupillometric data, the Eyelink 1000 eye tracker was used and pupil diameter was continuously recorded during the experiment, binocularly at 120 Hz. Participants were seated approximately 60 cm from the monitor.

Participants were asked not to do any heavy exercise or drink any caffeinated drinks 2–3 h prior to participation and to have a good night’s sleep involving at least 7–8 h sleep the night before. Prior to the day of the study, participants sent a 30-min long playlist with their preferred background tracks to the researcher. When completing the music condition background music was played continuously throughout the task from a mobile device in “do not disturb” mode to avoid any distractions. Participants could either use their own headphones or a pair provided by the researcher to listen to the music. For the music-absent condition, the headphones were removed to increase ecological validity (given that participants would not normally wear headphones when completing a task in silence).

Upon first arrival in the lab at the start of the experiment, participants first read the information sheet and signed the consent form. Their dominant eye was then calibrated with a 5-point calibration and tested throughout the experiment. They then received the task instructions and were presented with five practice trials before the first block in both the music-present and music-absent conditions. They had the opportunity to ask any questions throughout the practice trials.

The instructions they received for the practice trials also applied to the main task. First participants were instructed to look at the fixation cross in the centre of the screen before each trial. Then, it was explained to them that their tasks were (i) to stop the clock-hand in the clock-face in the middle of the screen as soon as the clock-hand started moving, by pressing the left mouse button; (ii) when presented with thought-probe descriptions after some of the trials, to choose the statement that best described their current conscious experience by pressing buttons 1, 2, or 3 on the keyboard.

The study took approximately 1 h and 30 min, during which time participants performed the sustained-attention task twice: once in silence (30 min) and once with their preferred music playing in the background (30 min). There were five blocks of trials in each condition. Each block was started individually by the experimenter, ensuring that there was a break for a few minutes between the blocks so that participants could rest and drink some water if they wished to. To control for carry-over effects of the music conditions, music conditions were counterbalanced and there was a 10-min break between the conditions in both orders. During the break, participants used a tablet to play a word spelling game called ‘Hi Words’ that was unrelated to the study. The game kept them engaged and helped them recover from the fatigue caused by the first condition (e.g., Jahncke et al., 2011).

Once participants had finished the task, the researcher recorded their age, gender and whether they had had any previous formal music training and, if yes, for how many years. Then, the participants received the debriefing sheet and £5 for their participation.

## Results

This study formed part of a larger study the first part of which was reported in Kiss and Linnell (2021) and which showed that background music decreased proportions of mind-wandering states, increased proportions of task-focus states, and did not affect external-distraction states. The aim of the current study was to explore the relationship between arousal as indexed by pupil responses - including pretrial pupil diameter and task-evoked pupillary responses (TEPRs) - and subjectively reported states of mind-wandering, task-focus, and external-distraction, both with and without background music. We also focused on exploring the link between arousal and background music and whether arousal mediates the effect of background music on attentional states. To examine all of these questions, we conducted similar analyses on both pretrial pupil diameter and TEPR.

Analyses on the pupil data were conducted similarly to those reported in Unsworth and Robison (2016). Data from each participant’s dominant eye were used and missing data points due to blinks, off-screen fixations, and eye-tracker malfunction were removed. Pretrial pupil diameters were computed as the average pupil diameter during the fixation period (the 2000 ms during which participants saw a fixation cross in the middle of the screen). Pretrial pupil diameters were z-score normalised for each participant to correct for individual differences. Outliers, counted as z-scored data points above 3 or below − 3, were excluded from analysis.

Similarly to Unsworth and Robison (2016), to examine how much the pupil dilates relative to baseline levels, TEPRs were calculated using the last 200 ms of the wait time as baseline and starting from when the clock hand started moving on a trial-by-trial basis for each participant. To examine the time course of the TEPRs, the pupil data for each trial were averaged into a series of 20 ms time windows following stimulus onset until the participant gave a response. For example, for trials with a wait time of 10 s, the baseline was defined as the average pupil diameter between 9.8 s and 10 s into the wait time. Then, TEPR was calculated for each 20 ms time window starting from 10 s, by subtracting the baseline diameter (i.e., the average pupil diameter calculated for the period between 9.8 s and 10 s into the wait time) from the average pupil diameter for each of the following 20 ms time windows. The maximum TEPR from across the 20 ms segments was then chosen for each participant and z-score normalised and used for the analyses.

In this section, first results from linear mixed model analyses are reported that examined the relationship between pretrial pupil diameter and attentional states, including task-focus, mind-wandering, and external-distraction states. We also report results from the ANOVA that examined that effect of music present/absent (music P/A) and time-on-task (blocks 1, 2, 3, 4, and 5) on pretrial pupil diameter. Next, results of a mediation analysis are reported exploring whether the effect of music P/A on the balance of mind-wandering and task-focus states is mediated by pretrial pupil diameter. Finally, we report analyses focusing on TEPRs including results of linear mixed models exploring the relationship between TEPRs and attentional states as well as results of an ANOVA analysis that was performed on the effect of music P/A and time-on-task on TEPR. Given that there were no effects of music P/A on TEPRs, no mediation analysis was performed for TEPRs.

### Pretrial pupil diameter

First, linear mixed models were conducted to explore the relationship between attentional-state category (mind-wandering, task-focus, external-distraction states) and pretrial pupil diameter (using pupil diameters for the single trials immediately after which participants reported the given attentional-state category). In the models, subjects were entered as random effects and attentional-state category as the fixed effect. First, on- and off-task states were compared. Results showed that on-task attentional states (i.e., task-focus states) were linked to significantly larger pupil diameter (*M* = 0.114, *SE* = 0.026) than off-task states (i.e., a combination of mind-wandering and external distraction states; *M* = − 0.082, *SE* = 0.031), *t* = -4.843, *p* < .001 (*b* = − 0.200, *SE* = 0.041).

**Fig. 3:**
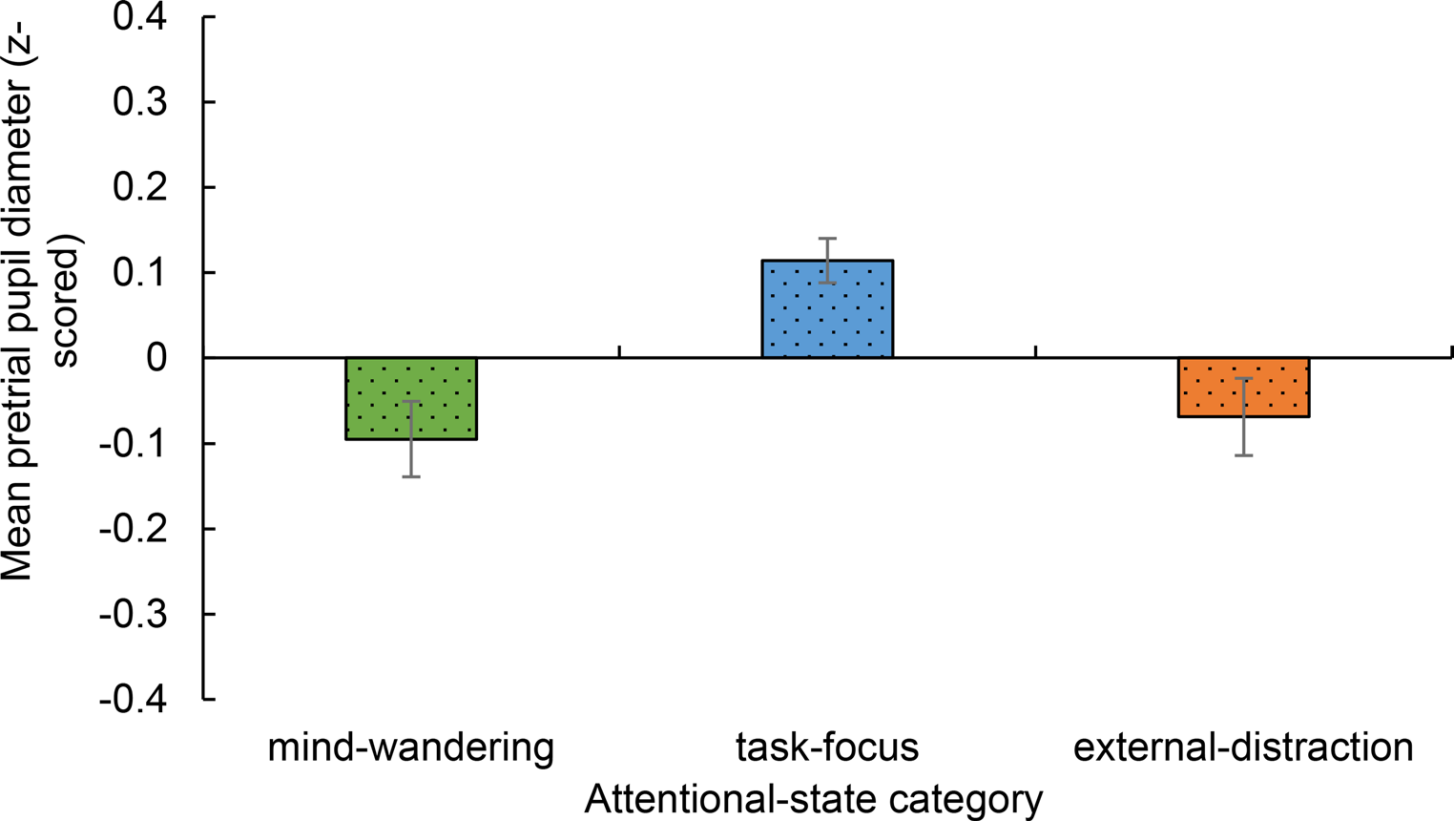
Mean pretrial pupil diameter (z-scored) as a function of attentional state category. Error bars represent ± 1 standard error of the mean (S.E.M.)

Examining all three attentional states separately, results showed a significant difference between task-focus and mind-wandering states, *t* = -4.098, *p* < .001 (*b* = − 0.209, *SE* = 0.051), and between task-focus and external-distraction states, *t* = 3.556, *p* < .001 (*b* = 0.183, *SE* = 0.052). As can be seen in Fig. 3, task-focus states were linked to significantly *larger* pretrial pupil diameter than mind-wandering or external-distraction states. There was no significant difference between mind-wandering and external-distraction states (*p* = .680).

**Fig. 4:**
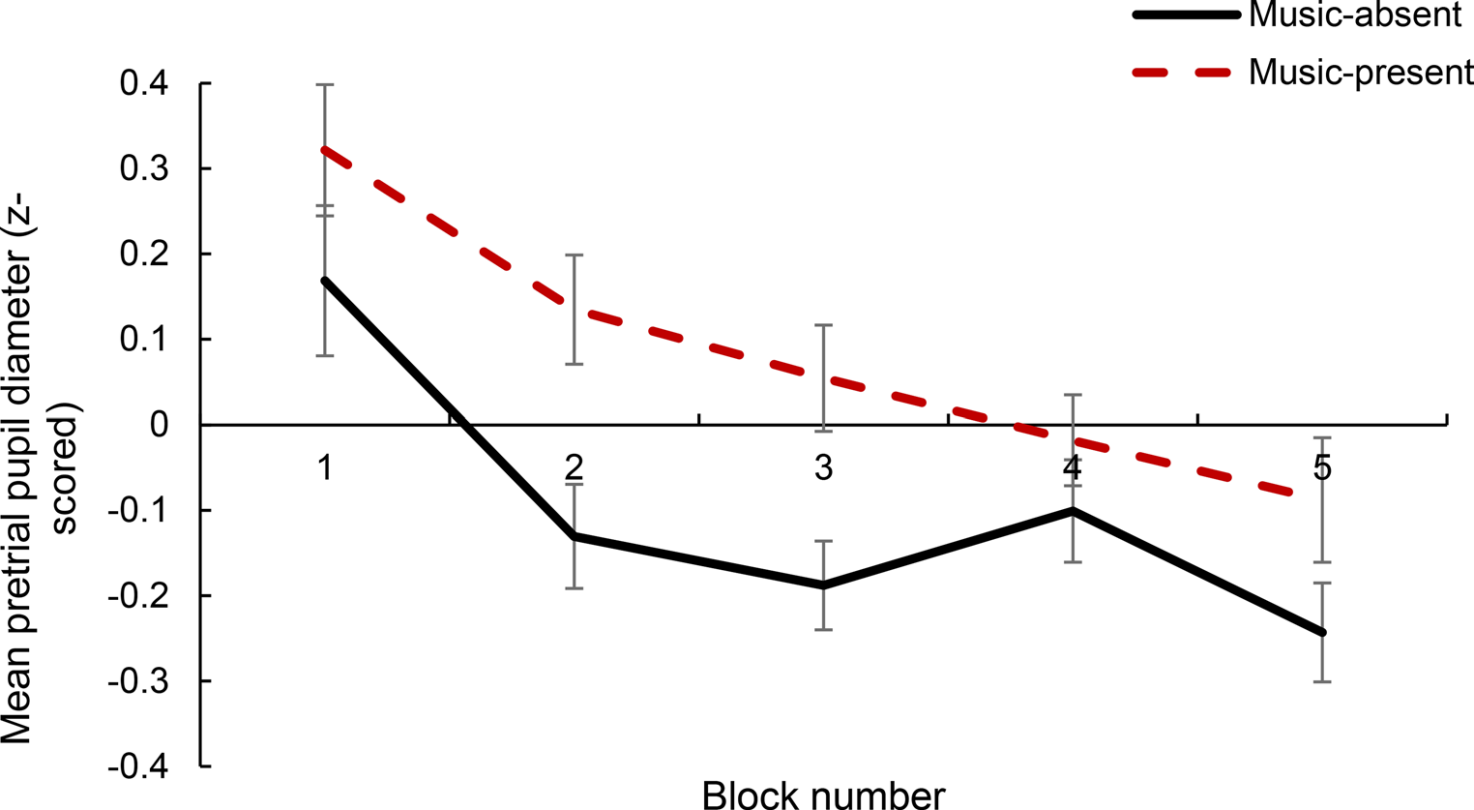
Mean pretrial pupil diameter (z-scored) as a function of time-on-task (block 1, 2, 3, 4, and 5) and music P/A. Error bars represent ± 1 S.E.M

Next, a 5 × 2 within-subjects factorial ANOVA was conducted to examine the effect of music P/A and time-on-task on z-score normalised pretrial pupil diameter. The ANOVA showed that there was a significant main effect of music P/A on pretrial pupil diameter, *F*(1, 38) = 4.711, *MSE* = 2.917 *p* = .036, *partial η2* = 0.110. Specifically, pretrial pupil diameter was significantly larger in the music-present condition (*M* = 0.078, *SD* = 0.949) than in the music-absent condition (*M* = − 0.094, *SD* = 0.960). The ANOVA also showed a significant main effect of time-on-task, showing that pretrial pupil diameter decreased with time-on-task, *F*(4, 98.952) = 12.908, *MSE* = 3.056, *p* < .001, *partial η2* = 0.254 (see Fig. 4). Post-hoc t-tests with Bonferroni correction comparing each block showed that there was a significant difference between blocks 1–2, 1–3, 1–4, 1–5 (*p* < .001).

There was no significant interaction between music P/A and time-on-task (*p* = .525, see Fig. 4).

**Fig. 5:**
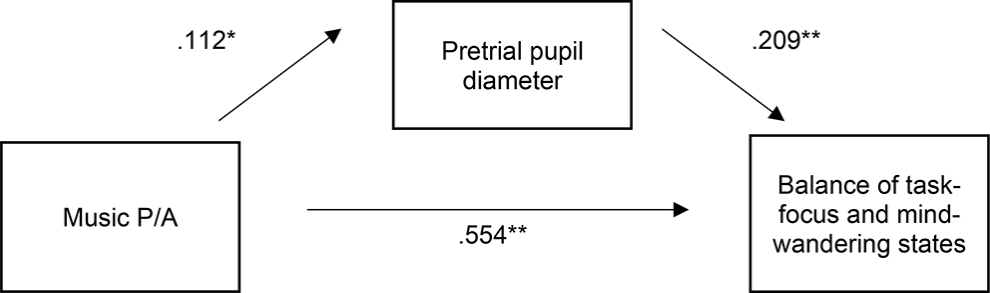
Regression coefficients between music P/A as the predictor, pretrial pupil diameter as the mediator, and task-focus versus mind-wandering states as the outcome variable. * *p* < .050. ** *p* < .001

Additionally, a mediation analysis was performed to investigate whether pretrial pupil diameter mediated the effect of music P/A on whether mind-wandering or task-focus states were reported (inputted as a binary outcome variable). The focus here was on mind-wandering and task-focus states only given that music P/A did not affect external-distraction states (as reported in Kiss & Linnell, 2021). We used the 4.0 version of the PROCESS macro (an observed variable ordinary least squares and logistic regression path analysis modelling tool; Hayes, 2022) with bootstrapping procedures for the indirect effects (unstandardised indirect effects were computed for each of 5000 bootstrapped samples). The mediation model showed that the direct effect of music P/A on the mediator variable pretrial pupil diameter was significant, *b* = 0.112, *t*(1844) = 2.478, *p* = .013, indicating that pretrial pupil diameter was larger in the music-present than in the music-absent condition. Secondly, the mediator variable pretrial pupil diameter had a significant direct effect on the outcome variable (mind-wandering versus task-focus states), *b* = 0.209, *Z*(2) = 3.782, *p* < .001, indicating that as pretrial pupil diameter increased, participants more frequently reported task-focus than mind-wandering states. Thirdly, the direct effect of music P/A on the outcome variable was also significant, *b* = 0.554, *Z*(2) = 5.158, *p* < .001, indicating that participants reported more task-focus and less mind-wandering states in the music-present than in the music-absent condition. Importantly, the indirect effect of music P/A on the outcome variable as mediated by pretrial pupil diameter was significantly different from zero, *IE* = 0.024, 95% lower limit *CI* = 0.004, 95% upper limit *CI =* 0.051, indicating that pretrial pupil diameter mediated the effect of music P/A on the balance of task-focus and mind-wandering states (see Fig. 5).

### Task-evoked pupillary responses (TEPRs)

We examined the relationship between TEPRs and attentional-state category using linear mixed models (by measuring pupil diameters for the singe trials immediately after which participants reported the given attentional-state category). In the models, subjects were entered as random effects and attentional-state category as the fixed effect. The models did not show any significant results (*p* = > 0.262), suggesting that TEPRs did not change as a function of attentional state.

**Fig. 6:**
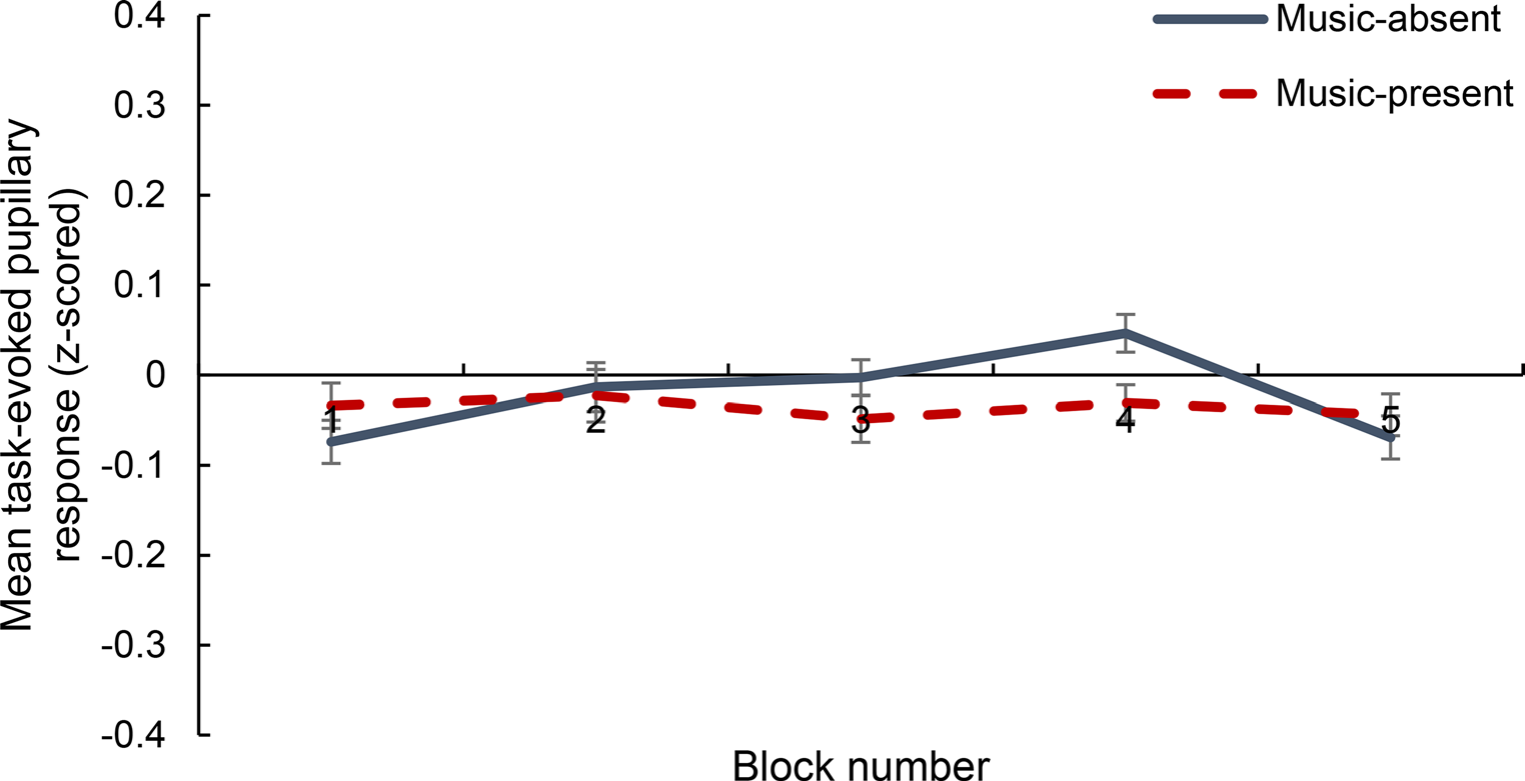
Mean task-evoked pupillary response (z-scored) as a function of time-on-task (block 1, 2, 3, 4, and 5) and music present/absent. Error bars represent ± 1 S.E.M

Furthermore, a 5 × 2 within-subjects factorial ANOVA was conducted to examine the effect of music P/A and time-on-task on z-score normalised TEPRs. The ANOVA showed no significant main effect of music P/A (*p* = .564) but a significant main effect of time-on-task *F*(4, 152) = 2.511, *MSE* = 0.053, *p* = .044, *partial η2* = 0.062 (see Fig. 6). Post-hoc t-tests with Bonferroni correction showed that there was a significant difference between TEPRs for blocks 1–4 (*p* = .003) and blocks 4–5 (*p* = .002).

The ANOVA also showed a significant interaction between music P/A and time-on-task, *F*(4, 152) = 2.449, *MSE* = 0.056, *p* = .049, *partial η2* = 0.061 (see Fig. 6). Post-hoc t-tests with Bonferroni correction showed that only in the music-absent condition did blocks 1–4 (*p* < .001) and blocks 4–5 (*p* = .002) significantly differ.

## Discussion

The aim of the present study was to build on the findings of Kiss and Linnell (2021) on the effect of self-selected or preferred background music on subjectively reported attentional states of mind-wandering, task-focus, and external-distraction. In a population of students between the ages of 19 and 32 who normally listen to background music, the study explored the effect of self-selected background music on arousal as indexed by pupil responses - namely pretrial pupil diameter and task-evoked pupillary responses (TEPRs) - and the link between arousal indexed in this way and the subjectively reported states of mind-wandering, task-focus, and external-distraction with and without background music. The present results showed that, as predicted, background music increased arousal, as indexed by pretrial pupil diameter. Also as predicted, there was a link between pretrial pupil diameter and subjectively reported attentional state but the nature of this link was not completely as expected from the findings of Unsworth and Robinson (2016), showing as it did that task-focus states were linked to higher arousal level than external-distraction or mind-wandering states. Importantly, pretrial pupil diameter was found to mediate the effect of background music reported in Kiss and Linnell (2021) to increase task-focus states and decrease mind-wandering states. This makes sense given the aforementioned findings that background music increased arousal and task-focus states were associated with higher arousal than mind-wandering states.

Concerning the link between arousal and attentional state, results showed that when participants reported being on-task (i.e., experiencing task-focus states) their pretrial pupil diameters were significantly larger than when they reported being off-task and experiencing either mind-wandering or external-distraction states. Results did not show a difference in pretrial pupil diameters linked to mind-wandering and external-distraction states. These results highlighting the difference between on- and off-task states are consistent with past work using attention-control tasks such as the Psychomotor Vigilance Task or Stroop Task and showing that off-task states are linked to smaller pretrial pupil diameter than task-focus states (e.g., Grandchamp et al., 2014; Mittner et al., 2014; Unsworth & Robison, 2016, 2017, 2018; Unsworth et al., 2018). The finding that mind-wandering states were linked to smaller pretrial pupil diameters than task-focus states is supported by findings by Unsworth and Robison (2016), similarly showing mind-wandering states to be linked to smaller pupil diameters than task-focus states. However, the finding that external-distraction states were also linked to smaller pretrial pupil diameters than task-focus states is not supported by the findings of Unsworth and Robison (2016) which showed that external-distraction states were linked to significantly larger pretrial pupil diameters than task-focus states.

Even though our finding that external-distraction states were linked to lower pretrial pupil diameter than task-focus states is not in-line with Unsworth and Robison (2016), it is in-line with the Lenartowicz model (Lenartowicz et al., 2013): this model highlights that the key distinction between mind-wandering and external-distraction states is not one of arousal but of whether the orientation of attention is internal or external, with mind-wandering states distinguished by an internal orientation and external-distraction states by an external orientation. Lenartowicz et al. (2013) distinguishes between low- and high-arousal forms of mind-wandering in the guise of *mind-wandering* and *racing thoughts* (see also Unsworth & Robison, 2018) and between low- and high-arousal forms of external-distraction in the guise of *prepotent responses* and *stimulus sensitivity*. In fact, it is suggested that whether the attentional states of mind-wandering and external-distraction are associated with sub-optimal or supra-optimal arousal can be determined by factors such as individual differences in baseline arousal, individuals’ propensity to shift to high-arousal states (i.e., stress) or low-arousal states (i.e., boredom) in response to completing a task, and the context in which the task is completed (Lenartowicz et al., 2013; Unsworth & Robison, 2016, 2018). Here we only involved participants who normally prefer listening to background music and they completed the sustained attention task not only in silence as in Unsworth and Robison (2016) but also with their chosen background music. Involving music in the study and recruiting participants who prefer listening to music to a study advertised to be about background music listening could have led to the experience of external-distraction states that were linked to sub-optimal arousal, as compared to the experience of external-distraction states linked to supra-optimal arousal reported by Unsworth and Robison (2016). The focus of the present study on people who listen to background music during attention-demanding tasks means that the results can only be applied to people who use background music in this way. Thus, future research should aim to generalise these findings by recruiting participants who do not necessarily engage in background music listening.

Similarly to Unsworth and Robison (2016), however, the present results showed that arousal as indexed both by pretrial pupil diameter and TEPRs decreased with time-on-task. This arousal decrease with time-on-task was not influenced by the presence of background music which was in-line with the behavioural findings reported in Kiss and Linnell (2021), not showing an effect of music on time-on-task increases in reaction time. The significant overall decrease in pupillary measures with time-on-task is consistent with past research showing that pupil diameter decreases with an increase in fatigue and decrease in arousal (e.g., Beatty, 1982; Morad et al., 2000; Unsworth & Robison, 2016; Wilhelm, Giedke, Lüdtke, Bittner, Hofmann, & Wilhelm, 2001). Similarly, it is consistent with research on the LC-NE showing that, as vigilance decreases, both pretrial LC-NE activity and task-evoked LC-responses decrease (e.g., Aston-Jones et al., 1994; Aston-Jones et al., 2007). Thus, the current results show that the pupillary measures used – both pretrial pupil diameter and TEPR – accurately tracked the vigilance decrement (i.e., the decrease in arousal and increase in fatigue with time on task) in a sustained attention task.

Although there were no time-on-task effects of music on arousal, as predicted, background music increased participants’ arousal level, as indexed by pretrial pupil diameter, compared to silence. The increase in arousal with music is in-line with findings reported in Kiss and Linnell (2021) showing that background music increased task-focus and decreased mind-wandering states, taking into account the findings just reported and work by Unsworth and Robison (2016), both linking mind-wandering states to lower arousal than task-focus states. Support for the arousal-increasing effect of music measured with pupillometry comes from both the background music literature (a Master’s thesis study conducted by Tamalinuaite, 2017) and from the broader music literature not specific to background music (e.g., Gingras et al., 2015). Notably, research highlights that when music is familiar (Jagiello et al., 2019) or liked (Bianco et al., 2019) there is even greater pupil dilation in response to music listening (when listening is not accompanied by the performance of a concurrent task). It was most likely the case that the music listened to in the current study was familiar and liked given that participants were asked to self-select the music tracks and to include tracks they normally listen to while performing attention-demanding tasks. This being the case, it makes sense that there was a significant increase in arousal during background music listening, as indexed here by an effect of music on pretrial pupil diameter, albeit not on TEPR. Nevertheless, although one can infer familiarity with and liking for the *self-selected* music excerpts in the current study, a limitation of the study is that these parameters of the music (i.e., liking for and familiarity with the chosen music excerpts) were not explicitly measured; to know for certain whether participants choose familiar, liked music to listen to, future studies should directly measure these parameters.

Given that music increased pretrial pupil diameter while also increasing task-focus and decreasing mind-wandering states, and given that task-focus states were linked to higher pretrial pupil diameter than mind-wandering states, we expected pretrial pupil diameter to act as a mediator between music and the balance of task-focus to mind-wandering states. As expected, pretrial pupil diameter indeed mediated the effect of background music on the balance of these attentional states, suggesting that the increase in arousal caused by the music led to a decrease in mind-wandering states and increase in task-focus states. A relevant theory here, that highlights the role of arousal, is the mood-and-arousal hypothesis which states that music listening leads to an increase in positive mood and arousal which in-turn increases task-performance (Husain et al., 2002; Thompson et al., 2001; Schellenberg, 2005; Schellenberg & Hallam, 2005; Schellenberg et al., 2007). Nonetheless, the mood-and-arousal hypothesis has been developed to explain the effects of music that is listened to in advance of a task rather than whilst it is being performed and there is less clear evidence for the mood-and-arousal hypothesis when music is listened to during task-performance, especially when the task is a simple attention-demanding task (Begum, Uddin, Rithy, Kabir, Tewari, Islam, & Ashraf, 2019; Cloutier et al., 2020; Du, Jiang, Li et al., 2020; Fernandez et al., 2020; Jiang et al., 2011; Mammarella et al., 2007; Marti-Marca et al., 2020; Nguyen & Ghan, 2017; Perham & Sykora, 2012; Shih et al., 2016). Furthermore, the mood-and-arousal hypothesis only predicts task-performance and does not predict effects of music on attentional state. The present result therefore adds to the discussion about the applicability of the mood-and-arousal hypothesis to performance with background music and extends the discussion to attentional state by showing that, during a simple sustained attention task, pretrial pupil diameter as an objective arousal measure mediates the effect of background music on attentional state. Here, however, the focus was exclusively on arousal and mood was not explicitly measured. Thus, future studies should explore whether mood, in addition to or in conjunction with arousal, mediates the effect of background music on attentional state.

## Conclusion

In sum, the present study aimed to explore, for the first time in the context of background music listening, the link between subjectively reported attentional states, namely mind-wandering, task-focus, and external-distraction states, and objectively measured arousal (measured with pretrial pupil diameter and task-evoked pupillary responses). This study builds on recent results showing an increase in task-focus states and decrease in mind-wandering states with preferred background music (Kiss & Linnell, 2021) and shows that there is a significant link between attentional states (i.e., mind-wandering, task-focus, and external-distraction states) and arousal in the context of background music listening. Importantly, results of this study showed that arousal mediated the effect of background music on the balance of mind-wandering and task-focus attentional states, suggesting that an increase in arousal caused by background music leads to decreases in mind-wandering, and increases in task-focus, attentional states.

## Data Availability

The datasets obtained during, and/or analysed during, the current study are available from the corresponding author on reasonable request.
